# Facultative symbionts are potential agents of symbiont-mediated RNAi in aphids

**DOI:** 10.3389/fmicb.2022.1020461

**Published:** 2022-11-23

**Authors:** Tong Li, Yongjun Wei, Chenchen Zhao, Shaojian Li, Suxia Gao, Yuanchen Zhang, Yuqing Wu, Chuantao Lu

**Affiliations:** ^1^Institute of Plant Protection, Henan Key Laboratory of Crop Pest Control/Key Laboratory of Integrated Pest Management on Crops in Southern Region of North China, Henan Academy of Agricultural Sciences, Zhengzhou, China; ^2^School of Pharmaceutical Sciences, Laboratory of Synthetic Biology, Zhengzhou University, Zhengzhou, China; ^3^Henan International Laboratory for Green Pest Control /College of Plant Protection, Henan Agricultural University, Zhengzhou, China; ^4^College of Biological and Food Engineering, Anyang Institute of Technology, Anyang, China

**Keywords:** insect bacteria, aphid symbionts, symbiont-mediated RNAi, *Buchnera aphidicola*, horizontal transmission

## Abstract

Aphids are major crop pests, and they can be controlled through the application of the promising RNA interference (RNAi) techniques. However, chemical synthesis yield of dsRNA for RNAi is low and costly. Another sustainable aphid pest control strategy takes advantage of symbiont-mediated RNAi (SMR), which can generate dsRNA by engineered microbes. Aphid host the obligate endosymbiont *Buchnera aphidicola* and various facultative symbionts that not only have a wide host range but are also vertically and horizontally transmitted. Thus, we described the potential of facultative symbionts in aphid pest control by SMR. We summarized the community and host range of these facultative symbionts, and then reviewed their probable horizontal transmitted routes and ecological functions. Moreover, recent advances in the cultivation and genetic engineering of aphid facultative symbionts were discussed. In addition, current legislation of dsRNA-based pest control strategies and their safety assessments were reviewed.

## Introduction

Aphids (Hemiptera: Aphididae) are tiny and soft-bodied insects. Over 5,000 aphid species have been identified globally, with roughly 10% of them being major agricultural and forest pests (Blackman and Eastop, 2017). In China, the harvest from 10 to 15 million hectares of wheat plantation is lost annually due to aphid infestations (Hu et al., 2016). Moreover, aphids are primary carriers of plant viruses, transmitting about half of all insect-vectored viruses (Ziegler-Graff, 2020). In addition, through their honeydew, aphids promote the growth of saprophytic ascomycetes on plants, resulting in sooty molds that hamper photosynthesis. Neonicotinoid pesticides are widely used to control aphids in agroecosystems. Due to their negative impact on the health and activity of pollinators (Henry et al., 2012; Tan et al., 2015; Tsvetkov et al., 2017), their use is restricted. Hence, more environmentally friendly aphid control techniques are of great interest.

In the revolutionary RNA interference technique (RNAi) (Bernstein et al., 2001; Bartel, 2004), double-stranded RNA (dsRNA) is delivered into host cells, triggering RNAi effects that ultimately inhibit the expression of the target gene. By specifically inhibiting the expression of essential genes in pests, RNAi is a potential pest management strategy, as it causes aberrant insect development and death (Vogel et al., 2018; Christiaens et al., 2020). Efficient delivery of intact and sufficient dsRNA into host cells and in aphids are essential for RNAi; this was realized by injection, topical application, and transgenic or artificial feed (Jaubert-Possamai et al., 2007; Abdellatef et al., 2015; Niu et al., 2019; Liu et al., 2021b). However, these delivery strategies are difficult to apply in aphid control, as they are labor-intensive and costly. Orally delivered dsRNA was degraded by aphid gut endonucleases (Christiaens et al., 2014; Ghodke et al., 2019). Bacterial symbionts of insects are potential producers and vectors of dsRNA (Guan et al., 2021). Through genetic engineering, these bacteria can be modified to constitutively synthesize and subsequently effectively deliver dsRNA within the pests, and this is referred to as symbiont-mediated RNAi (SMR) (Whitten et al., 2016).

Aphids have bacterial symbionts (Baumann, 2005), such as the obligate endosymbiont *Buchnera aphidicola* that supplies essential amino acids (Douglas, 1998). The co-evolution of this symbiosis is estimated to be 150–250 million years (Moran et al., 1993; Clark et al., 2000). Besides *B. aphidicola*, there exist other vertically transmitted symbiotic bacteria, and most are facultative symbionts (Russell et al., 2003). These facultative symbionts are potential SMR agents for the following reasons. Firstly, a few facultative symbionts are cultivable, and genetic engineering for dsRNA synthesis is possible. Secondly, facultative symbionts are not strictly associated with a specific host, enabling their infection of diverse aphid species. Thirdly, facultative symbionts are both vertically and horizontally transmitted and thus, dsRNA can be rapidly disseminated in an aphid population through genetically engineered strains.

The diversity of facultative symbionts of aphids and their occurrence were described, and the horizontal transmission routes and ecological functions of facultative symbionts in aphids potentially improved the dissemination of genetically modified symbionts in aphid populations were also discussed. Moreover, the cultivation and genetic engineering of facultative symbionts of aphids were highlighted. In this study, we reviewed recent advances of facultative symbionts as SMR agents for aphid control in insects.

## Symbiont-mediated RNAi in insects

The features of dsRNA synthesis methods and dsRNA delivered routes were summarized (Supplementary Table S1). As the chemical synthesis yield of dsRNA is low and costs high, microbe-mediated strategies are developed to replace it (Guan et al., 2021). Through feeding and injecting of engineered strains, RNAi has been successfully performed in diverse insects (Tian et al., 2009; Zhu et al., 2011; Li et al., 2011c; Zhang et al., 2012; Vatanparast and Kim, 2017; Ma et al., 2020). The ubiquity of insect symbiotic bacteria (McFall-Ngai et al., 2013) makes them potential dsRNA expression agents (Whitten and Dyson, 2017). Bacterial SMR was successfully applied in kissing bugs (*Rhodnius prolixus*), western flower thrips (*Frankliniella occidentalis*), and honeybees (*Apis mellifera*). *Rhodococcus rhodnii* is a facultative gut symbiont of *R. prolixus*, and it supplies vitamin B to the host (Baines, 1956; Pachebat et al., 2013). The *F. occidentalis* bacterial symbiont strain BFo2 is closely related to the *Pantoea* genus. The dsRNA-expressing *R. rhodnii* and Bfo2 strains effectively infected *R. prolixus* and *F. occidentalis*, respectively, and subsequently triggered systemic RNAi in their hosts (Whitten et al., 2016). *Snodgrassella alvi*, a gut bacterium symbiont of *A. mellifera*, was genetically engineered to trigger RNAi of certain pathways, and it activated an antiviral response in *A. mellifera*. Furthermore, by inhibiting the expression of mite genes, *S. alvi* strains were used to kill parasitic *Varroa* mites in *A. mellifera* (Leonard et al., 2020).

Insects lack RNA-dependent RNA polymerases (RdRp) to amplify dsRNA, and they could not sustain RNAi. Hence, high amounts of dsRNA are required to trigger RNAi in insects. In aphids, a dsRNA concentration of 1,500 ng/μL was used for plant-mediated RNAi (Ding et al., 2017; Shang et al., 2020a, b). In the meanwhile, the optimum concentration of dsRNA for RNAi was *ca.* 350 ng/μL for *Arabidopsis thaliana* (Dubrovina et al., 2019). SMR ensured persistent synthesis of dsRNA and triggered sustained RNAi in insects (Whitten and Dyson, 2017), which filled the RNAi gaps between lab-scale studies and field pest control.

## Facultative bacterial symbionts of aphids

### Discovery of facultative symbionts of aphids

**Figure 1:**
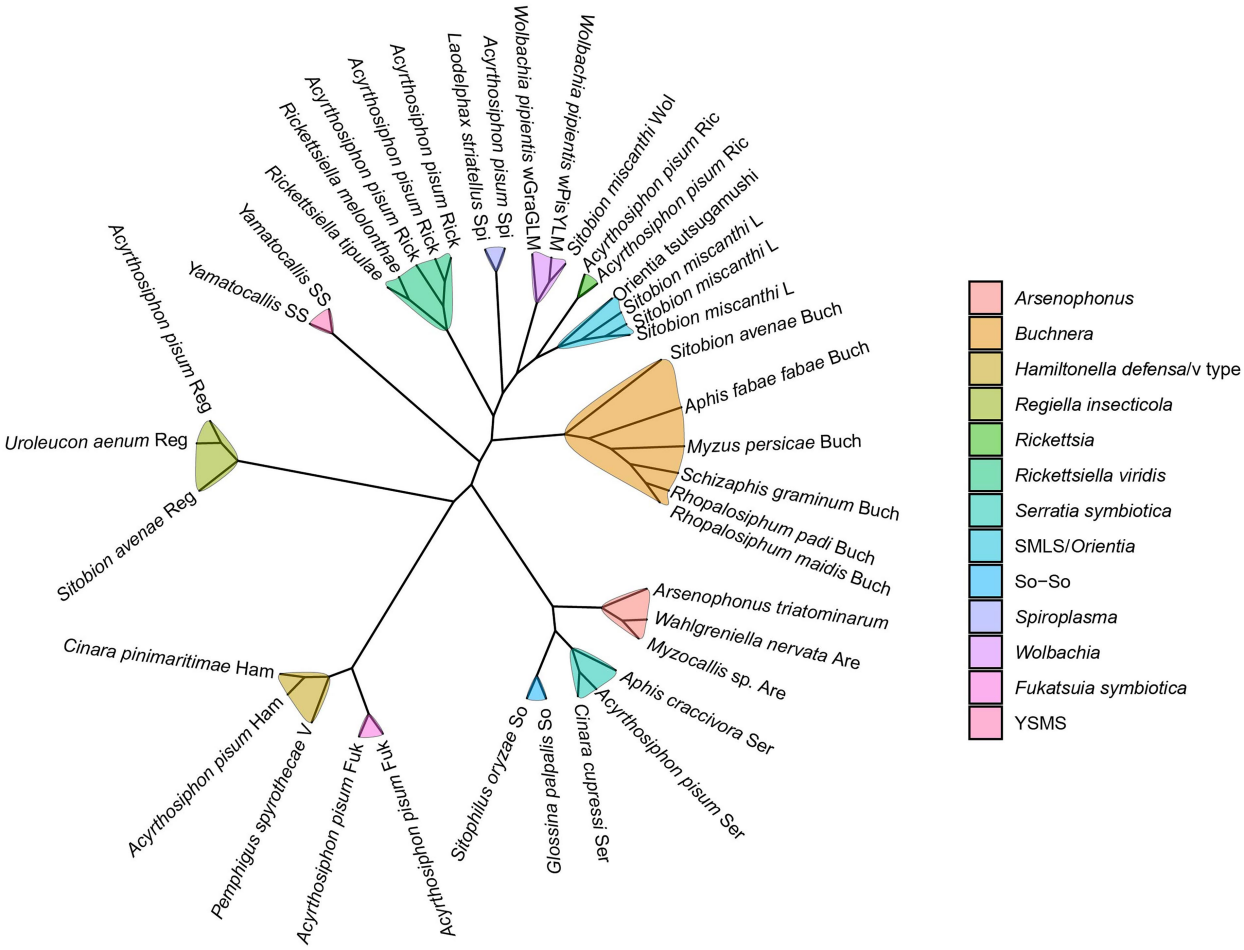
The cladogram of the aphid facultative symbionts. This cladogram is visualized by the ggtree R package (Yu et al., 2017). The information of the sequences used here is provided in the supplementary Supplementary Table S2.

Seven groups of known facultative symbionts are PAR (pea aphid *Rickettsia*) (Chen et al., 1996), PASS (pea aphid secondary symbiont, R type) (Chen and Purcell, 1997), PABS (pea aphid *Bemisia*-like bacterium, T type) (Darby et al., 2001), PAUS (detected in US pea aphid strains, U type) (Sandström et al., 2001; Tsuchida et al., 2002), PAXS (pea aphid X-type symbiont, X type) (Guay et al., 2009), YSMS (*Yamatocallis* secondary mycetocyte symbiont) (Fukatsu, 2001), and SMLS (*Sitobion miscanthi* L type symbiont) (Li et al., 2011b). PASS is phylogenetically closely related to *Serratia*, and is thus called “*Candidatus* Serratia symbiotica.” Likewise, PABS and PAUS were designated as “*Candidatus* Hamiltonella defensa” and “*Candidatus* Regiella insecticola,” respectively (Moran et al., 2005), and PAXS as “*Candidatus* Fukatsuia symbiotica” (Manzano-Marin et al., 2017). *F. symbiotica*, *H. defensa*, and *R. insecticola* formed a clade in the family Yersiniaceae of the order Enterobacteriales (Patel et al., 2019). SMLS is a new genus of Rickettsiaceae that is closely related to *Orientia tsutsugamushi* (Li et al., 2011a). Three other arthropod-related symbionts were detected in aphids, i.e., *Wolbachia* (Jeyaprakash and Hoy, 2000), *Spiroplasma* (Cisak et al., 2015), and *Arsenophonus* (Novakova et al., 2009). Based on phylogenetic analysis, two monophyletic clades, provisionally designated as V and So-So, are proposed for facultative symbionts of aphids (Russell et al., 2003; Figure 1).

### Host range of facultative symbionts of aphids

*S. symbiotica, H. defensa*, and *R. insecticola* are the three dominant facultative symbionts of aphids, and they have been detected in 74, 53, and 38 species, respectively (Zytynska and Weisser, 2016). The Macrosiphini Aphid group is closely associated with all these three symbionts (Sandström et al., 2001; Haynes et al., 2003). *Arsenophonus* was detected in 1/3 tested aphids from the *Aphis* genus (Jousselin et al., 2012) and was widely distributed in Hormaphidinae species (Xu et al., 2021b). *Wolbachia* was widespread in Aphididae isolated from China, and 109 of the 114 tested aphid species (112 belonging to Aphididae*)* had at least a *Wolbachia* strain (Wang et al., 2014). In the social aphid *Pseudoregma bambucicola*, *Wolbachia* was the dominant facultative symbiont (Liu et al., 2021a). Moreover, *Spiroplasma* and *Rickettsia* were detected in several *Aphis* species, including *A. craccivora* (Brady et al., 2014), *A. fabae* (Zytynska et al., 2015), *A. citricidus* (Guidolin and Consoli, 2017), and *A. aurantii* from China (Deng et al., 2019). *F. symbiotica* was detected in *Mollitrichosiphum* at a prevalence of less than 1% (Qin et al., 2021). Besides *S. miscanthi*, SMLS was also detected in the wheat-feeding aphids *Schizaphis graminum* and *Rhopalosiphum padi* (Li et al., 2011a). It is clear that these facultative symbionts have diverse distribution patterns in aphids, which is probably correlated to climatic and geographical factors (Tsuchida et al., 2002; Russell et al., 2003; Xu et al., 2021a).

## Transmission modes and ecological roles of facultative symbionts of aphids

### Transmission modes

**Figure 2:**
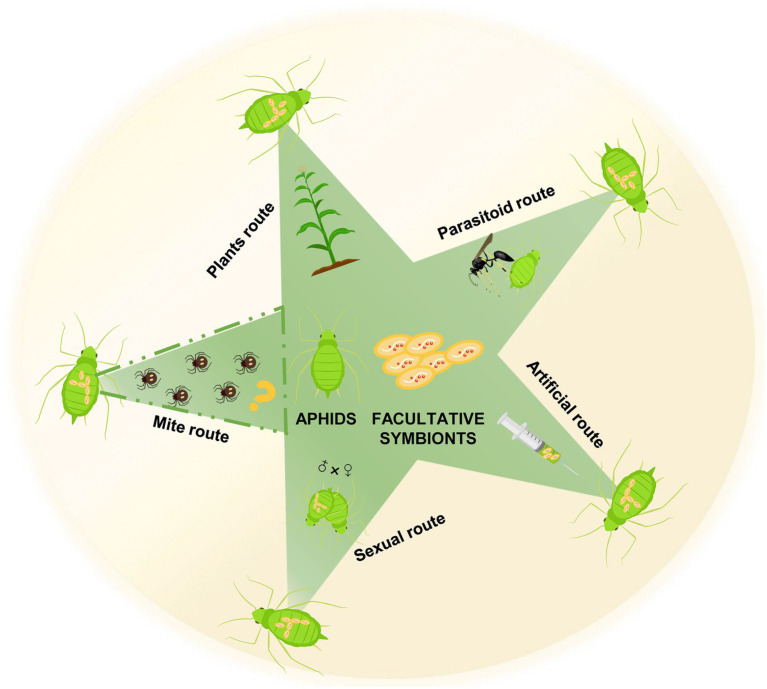
The potential horizontal transmission routes of aphid facultative symbionts. The question mark indicates that it is not clear whether aphid facultative symbionts can be horizontally transmitted by mites, though this route is often speculated.

The probable horizontal transmission routes of these facultative symbionts are diverse (Figure 2). Their vertical transmission efficiency was almost 100% (Fukatsu et al., 2000; Darby and Douglas, 2003; Li et al., 2011b). However, closely related facultative symbionts were detected in distantly related aphid hosts, implying horizontal transmission was available (Sandström et al., 2001; Russell et al., 2003; Li et al., 2014). Parasitoid wasps transferred facultative symbionts to uninfected aphids *via* contaminated ovipositors (Gehrer and Vorburger, 2012). Parasitoid transmission efficiency depended on the titer and haplotype of facultative symbionts (Kaech and Vorburger, 2020). *H. defensa* was detected in the honeydew and siphuncular fluid of infected aphids, indicating probable oral horizontal transmission (Darby and Douglas, 2003). Moreover, *H. defensa* and *S. symbiotica* were transmitted horizontally *via* plant feeding (Li et al., 2018; Pons et al., 2019a). Cultivated *S. symbiotica* strains were transmitted extracellularly *via* honeydew of infected aphids (Pons et al., 2019b), showing the high prevalence of *S. symbiotica* infections in ant populations after interactions with infected aphids (Renoz et al., 2019). Although ectoparasitic mites are vectors of bacterial symbionts in fruit flies (Jaenike et al., 2007; Brown and Lloyd, 2015), symbiont transmission in black bean aphids do not occur through mites (Gehrer and Vorburger, 2012). The paternal transmission of facultative symbionts occurred in pea aphids (Moran and Dunbar, 2006), with seldom sexual transmission between different lineages (Peccoud et al., 2014). This natural transmission is proposed in laboratory environments where microinjection tools are employed to artificially transmit facultative symbionts of aphids.

### Ecological roles

Temperature is a key environmental factor that affects aphids. *S. symbiotica* and *Rickettsia* infections increased the fitness of pea aphids under heat shock but decreased their fitness under normal temperature (Chen, 2000). *R. insecticola* and *F. symbiotica* infections increased the fecundity of aphids under heat stress (Heyworth et al., 2020). Parasitoid wasps and fungal pathogens are two key biotic factors affecting aphids. *H. defensa* infections conferred resistance to parasitoid attacks in aphids (Oliver et al., 2003). Moreover, the difference in levels of protection conferred by *H. defensa* strains was mainly due to co-infections by bacteriophages called APSEs (Brandt et al., 2017). *R. insecticola* infections increased the inclusive fitness of aphids under fungal attack by reducing the probability of fungal sporulation (Scarborough et al., 2005). Likewise, *Rickettsia*, *Rickettsiella,* and *Spiroplasma* spp. contributed to aphid resistance to fungal pathogens (Lukasik et al., 2012). Host specialization and body color are the main ecological characteristics of aphids. *R. insecticola* and *Arsenophonus* regulated the host usage in aphids (Tsuchida et al., 2004; Wagner et al., 2015). Specifically, *Arsenophonus* regulation was probably realized by changing the amino acid requirements in aphids (Tian et al., 2019). The endosymbiont *R. viridis* influenced prey–predator interactions by converting the body color of pea aphids from red to green (Tsuchida et al., 2010). Other endosymbionts affected the susceptibility of insects to insecticides (Kontsedalov et al., 2008; Ghanim and Kontsedalov, 2009; Liu and Guo, 2019). *S. symbiotica* infections increased the susceptibility of pea aphids to insecticides (Skaljac et al., 2018). Contrarily, in *S. miscanthi*, *H. defensa* infections decreased susceptibility to insecticides by probably increasing the activity of detoxifying enzymes (Li et al., 2021).
Interactions between aphids and facultative symbionts are complex. Apart from its facultative relationship with most aphid species, *S. symbiotica* form co-obligatory associations with *B. aphidicola* in members of Lachninae and Chaitophorinae (Lamelas et al., 2011; Manzano-Marin et al., 2017; Monnin et al., 2020). With the diversification of aphids, these dual symbiotic systems are replaced by those of distantly related bacterial symbionts (Manzano-Marin et al., 2017). Notably, a facultative symbiont can confer multiple fitness benefits to aphids. *F. symbiotica* like a “jack-of-all trades” symbiont, coinfect with other protective symbiont to provide resistance to parasitoid wasps, fungal pathogens, and heat stress (Heyworth and Ferrari, 2015). However, *F. symbiotica* infections cannot provide protective phenotype. These symbionts are more transmissible when other facultative symbionts infect the host (Doremus and Oliver, 2017). Furthermore, the protections conferred by facultative symbionts are costly. In the absence of stresses, these infections decrease aphid fitness (Gwynn et al., 2005; Vorburger, 2013; Kaech et al., 2022), hence, there exists a trade-off between the costs and benefits of facultative symbionts to aphids.

## Cultivation and genetic engineering of aphid facultative symbionts

Following long coevolution with their hosts, the genomes of insect bacterial symbionts reduce significantly, and genes are lost in all functional categories (Moran and Bennett, 2014). Empirically, current unculturable bacteria genomes are less than 0.5 Mb. *B. aphidicola* genomes are between 0.4–0.6 Mb, and they contain genes encoding the biosynthesis of essential amino acids for aphids. However, they lack genes involved in the biosynthesis of lipopolysaccharides and phospholipids, which are crucial for free-living bacteria (Shigenobu et al., 2000; Wernegreen and Moran, 2000; Moran and Mira, 2001; Tamas et al., 2002). This minimal set of genes in endosymbiotic bacteria necessitates stable host-supported environments as their natural habitats, making them highly fastidious. The genomes of *H. defensa* and *R. insecticola* are 2.11 Mb and 2.07 Mb (Degnan et al., 2009a, b), respectively, whereas that of *S. symbiotica* is 3.0 Mb (Burke and Moran, 2011). Unlike *B. aphidicola*, the genomes of these three symbionts retained genes encoding pathways for the biosynthesis of cell surface components, DNA repair, and bacterial secretion systems. Thus, they are more adaptable than *B. aphidicola* and culturable *in vitro* (Masson and Lemaitre, 2020). Indeed, *S. symbiotica* was the first aphid facultative symbiont, to be cultured on axenic media (Sabri et al., 2010), and was followed by *H. defensa* and *F. symbiotica* (Brandt et al., 2017; Patel et al., 2019), providing the possibility to engineer microbes for SMR.

To date, a few insect bacterial symbionts have been genetically engineered. Symbionts of leafhoppers (Arora et al., 2018), mosquitoes (Wang et al., 2017), and termites (Tikhe et al., 2016) were successfully transformed with plasmids containing exogenous genes. The bacterial symbionts of tsetse flies (Kendra et al., 2020; Keller et al., 2021), and honey bees (Powell et al., 2016; Leonard et al., 2018), were genetically engineered *via* genome editing. Recently, one of the aphid main facultative symbionts, *S. symbiotica*, is successfully modified with a variety of genetic techniques. Furthermore, the modified strain of *S. symbiotica* can be used to infect aphids, and induce the expressed heterologous genes within its host (Elston et al., 2020).

Facultative symbionts can infect various aphid tissues, and are detected in aphid hemolymph. Moreover, the facultative symbionts can horizontally transmit among aphids. These tissue tropism and transmitted patterns of facultative symbionts, would improve the spread of the dsRNA, and then trigger the systemic RNAi in aphids. The titer of the facultative symbionts is another concern with applying SMR in aphid. The populations of three aphid facultative symbionts, i.e., *S. symbiotica*, *Rickettsia*, and SMLS are estimated to reach more than 10^8^ per aphid (Koga et al., 2003; Sakurai et al., 2005; Li et al., 2011a). The populations of the SRM agents in *F. occidentalis* are estimated about 10^5^ per insect, hence, the concentrations of facultative symbionts are enough for the SMR in aphid. The titer of the engineered *S. symbiotica* in aphids, would reach an average of about 10^8^ colony-forming units (CFU) after proliferation for 5 days. The engineered facultative symbionts would also have strong colonization ability in aphids. Thus, genetically engineered facultative symbionts are possible strategies of SMR in the knockdown of aphid and plant virus genes for aphid control and paratransgenesis, respectively.

## The international policies on RNAi-based pest control strategies

In U.S., RNAi-based pest control products are divided into two types: RNAi in Plant-Incorporated Protectants (PIPs), and non-PIP dsRNA end use products (dsRNA-EPs). For the PIPs, the United States Environmental Protection Agency (US-EPA) will organize a scientific advisory panel (SAP) to consider aspects of risk assessment, and evaluate whether the examined PIPs can meet the environmental safety standard of the Federal Insecticide Fungicide and Rodenticide Act (FIFRA). In the dsRNA-Eps, dsRNA is viewed as biochemical by US-EPA, and the approach used to assess the ecological risks of traditional chemical pesticides (conventional chemicals and biochemicals) are used as a basis for ecological risk assessment. Furthermore, according to the title 40 of the Code of Federal Regulations (CFR) part 158 enacted by EPA, dsRNA-Eps are required to provide evaluation about their pesticide active ingredients and products. The details for the legislation of RNAi-based pesticide in U.S. can further referred in the white paper released by US-EPA in 2013 (EPA, 2013).

In the European Union (EU), any plant protection product (PPP) needs an authorization before commercialization. The authorization process is divided in approval of the active substance and in the authorization of the PPP itself. An approval of the active substance is the precondition for the authorization of a PPP. In the EU, PPPs are divided into chemicals or microorganisms, and a biopesticide category is not available. Hence, PPPs based on dsRNA are seen as chemical PPPs (Dietz-Pfeilstetter et al., 2021). The European Food Safety Authority (EFSA) organized the risk assessments of active PPP substance. The guidance documents produced by OECD (Organization for Economic Cooperation and Development), EPPO (European and Mediterranean Plant Protection Organization), and EFSA complement the regulations of PPPs. In 2020, OECD released a report of the considerations for environmental risk assessment of the application of sprayed or externally applied dsRNA-based pesticides (OECD, 2020), which detailed the safety assessments of RNAi-based pesticides in EU.

In 2018, the New Zealand Environmental Protection Authority announced that eukaryotic cells or organisms treated with dsRNA were not new organisms, and the risk assessments of such treatments in the open environment were unnecessary (EPA, 2018). Thereafter, the New Zealand Ministry of Primary Industries classified RNA in the “Negligible Risk Register” (MPI, 2018). The regulations of dsRNA-based pest control strategies are easy in New Zealand, which will probably promote the developments of such strategies. However, the native New Zealand biosafety researchers reminded that out of the regulations of dsRNA treatments would probably cause potential environmental risks (Heinemann, 2019).

## The safety assessment of RNAi-based pest control strategies

RNAi-mediated pest control strategies are the important parts of the future integrated pest management (IPM) system (Christiaens et al., 2020). To date, the risk assessment of RNAi-based genetically modified (GM) crops have been well documented. In EPA, 2013, US-EPA drafted a risk assessment guidance of RNAi-based crops (EPA, 2013). Subsequently, a scientific international workshop was organized by EFSA to evaluate the risk assessment of RNAi-based GM crops (European Food Safety Authority, 2014). As no new protein is generated by the RNAi-based GM crops, the safety assessment of RNAi-based products in EU, may not be complex as the transitional transgenic plants (Nitnavare et al., 2021). *Diabrotica virgifera* is a major pest during corn planting. In 2016, the commercialization of the first RNAi-based GM crop, MON87411, in the control of *D. virgifera*, was approved by Canadian Food Inspection Agency (CFIA), and then approved by US-EPA in 2017 (Zotti et al., 2018). In 2018–2021, EFSA evaluated the safety of MON87411 with other GM corns for food and feed uses. EFSA concluded that MON87411 was safe, and no safety concern was identified (EFSA Panel on Genetically Modified Organisms (GMO) et al., 2018, 2019, 2021). In 2021, the GMO safety certificate of MON87411 was approved by Ministry of Agriculture and Rural Affairs, PRC.

For human, insect bacterial symbionts are the friendly RNAi agents in pest control, as human have been exposed to insect bacterial symbionts for 1,000s of years (Popovici et al., 2010). To date, aphid facultative symbionts have only been identified in aphid species and their close insect relatives, such as whitefly (Darby et al., 2001; Bing et al., 2013). Although few studies evaluate the safety of aphid facultative symbionts to human, the systemic safety assessments of *Wolbachia* are referential. *Wolbachia* is the most widespread bacterial symbiont in insects, and has been used in the control of arboviruses transmission in *Aedes* mosquitoes. The safety assessments indicate that no *Wolbachia* related antigen is injected into the human volunteers, even after 1,000s of bites of *Wolbachia* infected mosquitoes (Popovici et al., 2010).

Increasing the specificity of dsRNA and reducing the off-target effects would strongly improve the safety of aphid SMR. The pest genomic information obtained from Genbank and the other insect genome sequencing project, such as i5k initiative, would effectively help us design aphid specific target sequences (Taning et al., 2021a, b). In the aphid SMR, employing aphid specific facultative symbiont as agents would provide a double insurance to avoid disturbing gene expressions in the non-target organisms (NTOs). Moreover, dsRNA is an environment friendly material, and the natural form of dsRNA is rapidly degraded in soil and aquatic systems within 2–7 days (Parker et al., 2019; Bachman et al., 2020). Additionally, the novel bacterial encapsulation methods would mitigate the environmental risk in aphid SMR (Arora et al., 2015).

However, some potential ecological risks should be noticed during the use of facultative symbionts in the aphid SMR. For instance, some *H. defensa* strains confer resistance to parasitoid attacks in aphid, hence, using of them as RNAi agents would negatively affect the biological control of parasitic wasps on aphids. Bacteriophages are known to be the key factor response to the parasitoid protection conferred by *H. defensa* (Brandt et al., 2017), using the bacteriophages negative *H. defensa* strains would avoid providing the parasitoid protection to aphids (Chuche et al., 2017). Furthermore, some species belonging to the same genus of aphid facultative symbionts are identified to be the plant pathogens (Salar et al., 2010; Bressan et al., 2012; Bressan, 2014; Dittmer et al., 2021). For aphids, the members of *Spiroplasma*, *Arsenophonus*, and *Rickettsia* are not recommended as the agents in SMR (Chuche et al., 2017).

## Conclusions and future perspectives

In addition to nucleic and mitochondrial DNA, facultative symbionts are thought to be the third form of genetic material in aphids. In contrast to *B. aphidicola*, aphid facultative symbionts can infect diverse aphid tissues and species, and they are horizontally transmitted within aphid populations. Although *in vitro* cultivation of the symbionts was previously challenging, recent breakthroughs in culture of aphid facultative symbionts will undeniably enhance research studies in their usage. In the future, proper engineered symbionts can be potential microbial agents for aphid control *via* SMR, which would help in improving crop growth.

## Author contributions

TL, YWe and CL conceived the study. TL, CZ, SL, SG and YZ drafted the manuscript. TL prepared the figures. CZ, SL and YWu revised the manuscript. TL, YWe and CL designed the whole study and revised the manuscript. All authors read, revised, and approved the manuscript.

## Funding

This work was supported by Henan Province Key R&D and Promotion Project (grant no. 222102110317), Henan Provincial Science and Technology R&D Program Joint Fund (grant no. 222301420107), Major public welfare scientific research project of Henan Province (grant no. 201300111600), National Natural Science Foundation of China (grant no. 31601897), and Fund for Distinguished Young Scholars from Henan Academy of Agricultural Sciences (grant no. 2020JQ05).

## Conflict of interest

The authors declare that the research was conducted in the absence of any commercial or financial relationships that could be construed as a potential conflict of interest.

## Publisher’s note

All claims expressed in this article are solely those of the authors and do not necessarily represent those of their affiliated organizations, or those of the publisher, the editors and the reviewers. Any product that may be evaluated in this article, or claim that may be made by its manufacturer, is not guaranteed or endorsed by the publisher.

## Supplementary material

The Supplementary material for this article can be found online at: https://www.frontiersin.org/articles/10.3389/fmicb.2022.1020461/full#supplementary-material
