# Supplementary material for: Facultative symbionts are potential agents of symbiont-mediated RNAi in aphids
Source: Front Microbiol. 2022 Nov 23;13:1020461. doi: 10.3389/fmicb.2022.1020461 (PMC9727308; doi:10.3389/fmicb.2022.1020461)
Supplement: Supplementary file 1 [file Table_1.docx]

Table S1. The summary of dsRNA synthesis methods and dsRNA delivery routes

| dsRNA synthesis methods | dsRNA delivery rotes | | | | | Features | | | | | References |
| --- | --- | --- | --- | --- | --- | --- | --- | --- | --- | --- | --- |
|  | Injection | Feeding | Spraying | Soaking | Topical delivery^a^ | Labor demand | Mass production^b^ | Cost^c^ | Applications | Commercialization^d^ |  |
| Chemical Synthesis | √ | √ | √ | √ | √ | Intensive | Hard | High | Indoor | No yet | (Mu et al., 2018) |
| Plant Expression |  | √ |  |  |  | Intensive | High | Medium | Indoor or in field | Yes | (Li et al., 2022) |
| Microorganism Expression | √ | √ | √ | √ |  | Less intensive | High | Low | Indoor or in field | No yet | (Guan et al., 2021) |

^a^, Topical application is mostly performed in the tiny insect groups, such as aphids; ^b^, the dsRNA scalability of chemical synthesis is estimated in milligram level, and that for microorganism fermentation is kilogram level; ^c^, dsRNA produced with chemical of platforms is about $100 000/g, dsRNA produced with encapsulated bacteria by RNAgri company (former APSE) is about $2/g (Maxwell et al., 2019); ^d^, Commercialization of dsRNA expression corn MON87411 has been approved in U.S., European Union and China.

References

Guan, R., Chu, D., Han, X., Miao, X., and Li, H. (2021). Advances in the development of microbial double-stranded RNA production systems for application of RNA interference in agricultural pest control. *Front. Bioeng. Biotechnol.* 9:753790. doi: 10.3389/fbioe.2021.753790

Li, X., Liu, X., Lu, W., Yin, X., and An, S. (2022). Application progress of plant-mediated RNAi in pest control. *Front. Bioeng. Biotechnol.* 10:963026. doi: 10.3389/fbioe.2022.963026

Mu, X., Greenwald, E., Ahmad, S., and Hur, S. (2018). An origin of the immunogenicity of in vitro transcribed RNA. *Nucleic Acids Res.* 46, 5239–5249. doi: 10.1093/nar/gky177
